# The intrinsic spatiotemporal structure of cognitive functions inspires the intervention of brain functions

**DOI:** 10.3389/fneur.2025.1494673

**Published:** 2025-02-13

**Authors:** Yifeng Wang, Chi Zhang, Qiang Liu, Xiujuan Jing

**Affiliations:** Institute of Brain and Psychological Sciences, Sichuan Normal University, Chengdu, China

**Keywords:** intrinsic spatiotemporal structure, precise intervention, spectral fingerprint, digital twin, brain stimulation

## 1 Introduction

**Figure 1:**
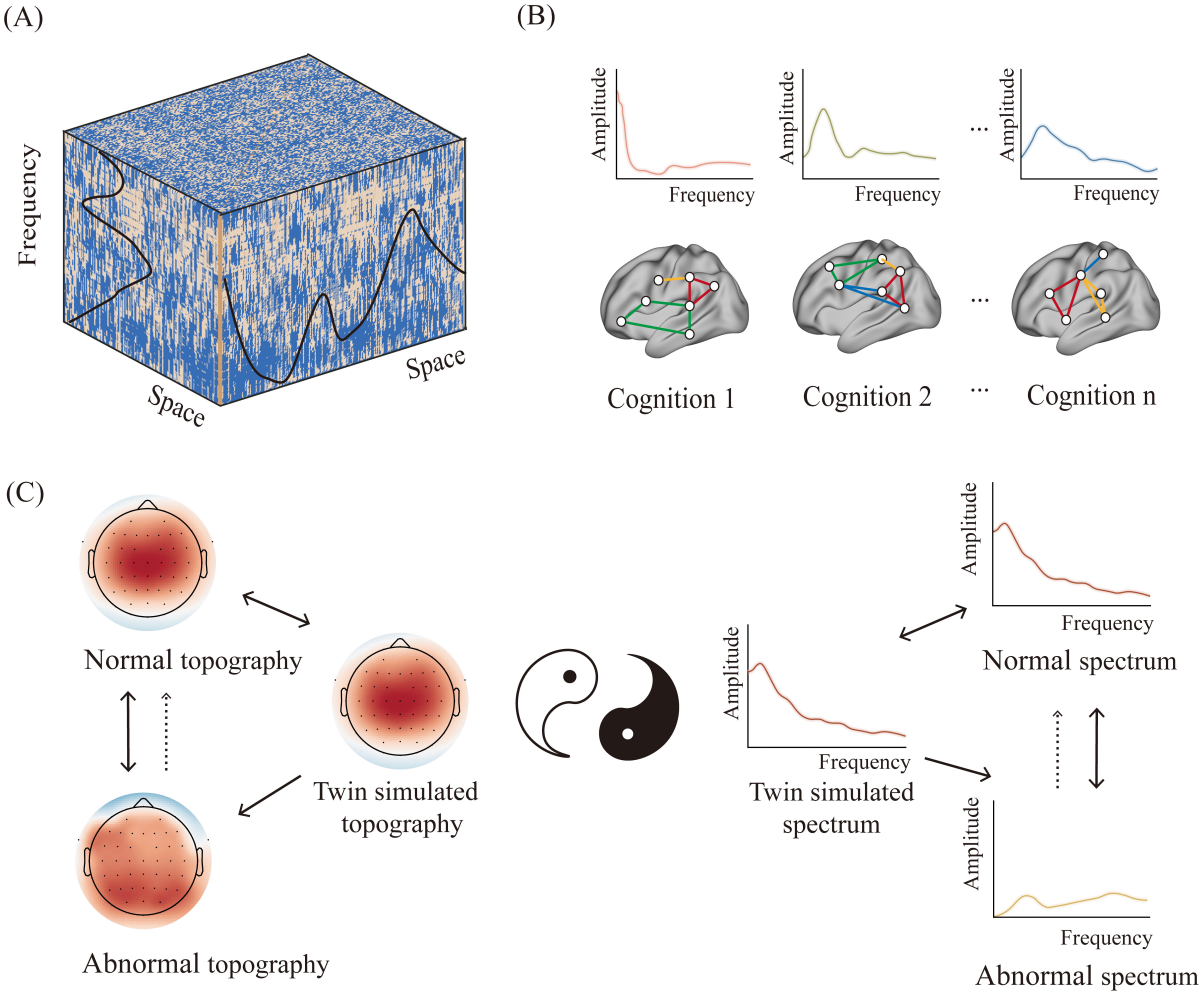
The intrinsic spatiotemporal structure of cognition and its implication for the intervention of brain functions. **(A)** The cognitive-specific spatiotemporal brain structure. **(B)** Cognitive fingerprints defined by specific neural circuits and power spectra. **(C)** Intervening in abnormal brain functions through the simulated twin spatiotemporal structure of normal brain functions.

All activities of entities occur within specific temporal and spatial scales. These scales are mutually restrictive. The spatiotemporal scales of neural activity are subject to the structural and functional organization of the brain. There is a strong connection between the size of the brain and the frequency of neural activity (1), while each brain region or neural circuit has its own spectral characteristics (2) (see Figure 1A). Consequently, cognitive-specific spectral fingerprints should be present if each cognitive function is realized by a distinct neural circuit. This phenomenon is elucidated by the spectral fingerprint hypothesis of cognition (3) (see Figure 1B). Recent studies have not only uncovered the frequency characteristics of specific brain regions or neural circuits but also shown that each cognitive process fluctuates at a preferential frequency (4).

In specificity, if a comprehensive cognitive function is divided into distinct cognitive processing stages, each associated with different neural circuits, then this cognitive function can be characterized by the spatiotemporal structure of nested neural circuits and corresponding spectral profiles. Assessing the level achievable by each cognitive function depends on the resemblance between the spatiotemporal structure of its neural circuit and the optimal spatiotemporal structure. Therefore, the intrinsic spatiotemporal structure of cognition is crucial for understanding the neural mechanisms that govern normal and pathological cognitive processes, as well as for cognitive rehabilitation and interventions in brain function.

## 2 Brain stimulation based on the intrinsic spatiotemporal structure of cognitions

By engaging in long-term cognitive training, individuals can optimize their cognitive abilities and gradually stabilize the neural circuits associated with those cognitive abilities, resulting in the emergence of specific spatiotemporal structures (5). The spatiotemporal structure of the neural circuit associated with optimal cognitive functioning can be defined as the intrinsic spatiotemporal structure of that cognitive function. Young adults, prodigies, or individuals with professional training often provide models that closely approximate the intrinsic spatiotemporal structure (6). Although many cognitive functions may appear similar at first glance, they are difficult to transfer from one to another due to their exclusive spatiotemporal structure. Different cognitive functions may share many brain regions, networks, and neural oscillations. However, this overlap is insufficient to produce transfer effects across cognitive functions after cognitive training. A specific cognitive function is defined by the complete spatiotemporal structure formed by both shared and non-shared components. Due to differences in intrinsic spatiotemporal structures, each cognitive function is unique, which may be a key reason for the lack of far-transfer effects in cognitive training. We refer to this phenomenon as cognitive isolation. Similar to productive isolation, cognitive isolation impedes the transition between two distinct cognitive functions. This may be a key reason for the absence of far-transfer effects in cognitive training (7).

The intrinsic spatiotemporal structure of cognitive functions may provide advantageous targets for brain stimulation. Current interventions for brain function are limited in their efficacy as they are restricted to non-specific neural circuits or frequencies, making it difficult to achieve an ideal brain state (6). On the other hand, interventions that target specific spatial or frequency elements of brain function have yielded remarkable outcomes (8). Therefore, it is exciting to anticipate the potential outcomes of brain stimulation techniques that are based on intrinsic spatiotemporal structures. In other words, achieving the optimal frequency of activity in each neural circuit, along with their interactions, may lead to the attainment of optimal cognitive function.

With the advancement of digital twin technology, it may become possible to mirror the twin spatiotemporal structure of every cognitive function in the future. According to the neural entrainment theory, this simulated twin spatiotemporal structure may cause the neural system to resonate in specific patterns (9), thereby optimizing the corresponding cognitive functions (see Figure 1C). It is encouraging that efforts have been made to combine high-definition transcranial electrical stimulation with broadband or band-removed spectral profiles, yielding favorable outcomes (10). This has promising implications for brain function rehabilitation and cognitive enhancement.

## 3 Discussion

The implementation of precision interventions is pivotal in improving the efficacy of non-invasive brain stimulation techniques. As each cognitive function is tied to specific neural circuits, stimulating a neural circuit at its intrinsic frequency inevitably yields more precise regulation of that cognitive function. However, when multiple cognitive processes or neural circuits are impaired, as commonly observed in mental disorders, the regulation of a single circuit or frequency may not necessarily achieve the desired outcome. Similarly, to enhance holistic cognitive functions, rather than focusing solely on improving a single stage of cognitive processing, it is essential to coordinate multiple circuits at various frequencies. In this case, the intrinsic spatiotemporal structure of cognitive functions can provide a more optimal solution.
